# Mortality Risk Factors and Survival Outcomes in Infants with Persistent Pulmonary Hypertension of the Newborn

**DOI:** 10.3390/jcm14134502

**Published:** 2025-06-25

**Authors:** Kokaew Chuaikaew, Gunlawadee Maneenil, Anucha Thatrimontrichai, Supaporn Dissaneevate, Manapat Praditaukrit

**Affiliations:** Division of Neonatology, Department of Pediatrics, Faculty of Medicine, Prince of Songkla University, Songkhla 90110, Thailand; ckokaew@medicine.psu.ac.th (K.C.); tanucha@medicine.psu.ac.th (A.T.); dsupapor@medicine.psu.ac.th (S.D.); manapat.p@psu.ac.th (M.P.)

**Keywords:** inhaled nitric oxide, neonatal intensive care, neonate, oxygenation index, pulmonary hypertension, risk factor, survival

## Abstract

**Background/Objectives**: Persistent pulmonary hypertension of the newborn (PPHN) is characterized by increased pulmonary vascular resistance, resulting in severe hypoxemia. This study determined the factors associated with increased risk of mortality and survival rate in infants with PPHN. **Methods**: This retrospective study was conducted between 2010 and 2023. The risk factors for mortality were assessed by Cox’s proportional hazard models, and the Kaplan–Meier survival curve was used to analyze the survival rates. **Results**: This study included 233 neonates with PPHN. Gestational age (GA) less than 28 weeks (adjusted hazard ratio [AHR] = 5.46, 95% confidence interval [CI]: 2.25–13.24, *p* < 0.001), Small for gestational age (SGA) (AHR = 2.93, 95% confidence interval [CI]: 1.24–6.92, *p* = 0.026), acute kidney injury (AKI) (AHR = 2.48, 95% CI: 1.27–4.84, *p* = 0.01), pneumothorax (AHR = 3.03, 95% confidence interval [CI]: 1.48–6.21, *p* = 0.003), vasoactive-inotropic score (VIS) at 24 h of age (AHR = 1.0026, 95% confidence interval [CI]: 1.0004–1.005, *p* = 0.026), and score for neonatal acute physiology II (SNAP-II) ≥ 43 (AHR = 4.03, 95% CI: 1.66–9.77, *p* = 0.005) were associated with an increased risk of mortality. The overall survival rate was 82.4%; it rose from 63.8% to 87.1% after inhaled nitric oxide (iNO) and extracorporeal membrane oxygenation (ECMO) were introduced (*p* < 0.001). The cumulative survival rates at the end of the 30 days were 62.1% (95% CI: 49.0–78.7) in the Pre-iNO era and 87.5% (95% CI: 82.7–92.6) in the Post-iNO/ECMO era, respectively (*p* < 0.001). **Conclusions**: GA less than 28 weeks, SGA, AKI, pneumothorax, high VIS and SNAP-II scores were associated with mortality in infants with PPHN. The improvement in the survival rate was related to the provision of advanced care, including iNO and ECMO therapy.

## 1. Introduction

Persistent pulmonary hypertension of the newborn (PPHN) occurs following the failure of postnatal adaptation of the fetal circulation, leading to a sustained elevation of pulmonary vascular resistance (PVR) [1]. Pulmonary blood flow diminishes and shunts through the fetal channels, resulting in hypoxemia [1,2]. The incidence of PPHN varies in different regions (0.43–6.82 per 1000 live births) [3]. PPHN can be either primary or secondary [4]. Common causes of PPHN are the maladaptation of pulmonary vasculature due to meconium aspiration syndrome (MAS), transient tachypnea of the newborn (TTN), and congenital diaphragmatic hernia (CDH) [5,6]. The mainstay of therapy in patients with PPHN is to reduce PVR and treat the underlying cause [7]. Therapeutic modalities for PPHN treatment include inhaled nitric oxide (iNO), pulmonary vasodilators, and extracorporeal membrane oxygenation (ECMO) [8,9]. The management of PPHN has advanced; however, the mortality of infants with PPHN is 20–35% [10,11]. Survivors may experience long-term adverse outcomes [4]. Evaluation of mortality-associated risk factors would be helpful in optimizing care and predicting the prognosis of patients with PPHN. Therefore, this study determined the factors associated with an increased risk of mortality and survival rate in infants with PPHN.

## 2. Materials and Methods

### 2.1. Setting and Population

This retrospective study was conducted in the NICU of Songklanagarind Hospital, a teaching hospital affiliated with the Prince of Songkla University, Thailand, between January 2010 and October 2023. Infants diagnosed with PPHN, including both inborn and outborn patients, were included in this study. The exclusion criteria were patients with chromosomal abnormalities, multiple anomalies, congenital heart disease, and CDH.

This article is a revised and expanded version of a paper entitled Survival of Infants with Persistent Pulmonary Hypertension of The Newborn: 12-year Experience, which was presented at the 9th Congress of the European Academy of Pediatric Societies (EAPS 2022) Conference, Barcelona, Spain, 7–11 October 2022 [12].

The Institutional Review Board and Ethics Committee of the Faculty of Medicine, Prince of Songkla University, Songkhla, Thailand, approved the study protocol (REC 62-059-1-1).

### 2.2. Data Collection

Data were collected from medical records. Data analysis included neonatal demographic data, underlying maternal disease and antenatal care complications, timing of PPHN diagnosis, causes of PPHN, and medical treatment. Arterial blood gas results and oxygen saturation (SpO_2_) levels were reviewed during the treatment period. The oxygen index (OI), vasoactive-inotropic score (VIS), and score for neonatal acute physiology II (SNAP-II) were assessed and recorded as parameters for predicting mortality. We evaluated the maximum value of the OI, VIS, and SNAP-II at 0–12, 12–24, 24–48, and 48–72 h of age.

### 2.3. Definitions

The diagnostic criteria for PPHN were a history of respiratory distress, refractory hypoxemia, and different cyanosis between pre-ductal and post-ductal sites. Echocardiography was performed to confirm the diagnosis by neonatologists or pediatric cardiologists. Increased pulmonary pressure, including right-to-left shunt or bidirectional shunt across the foramen ovale (FO) or ductus arteriosus (DA), was observed [3]. Systolic pulmonary artery pressure was estimated based on the peak velocity of tricuspid regurgitation, with the use of the modified Bernoulli equation. The direction of shunting through the ductus arteriosus was assessed using a high parasternal ductal view and categorized as left-to-right, bidirectional, or right-to-left [1].

The OI was calculated using the following formula: OI = FiO_2_ × mean airway pressure [MAP] × 100/PaO_2_ [13]. Inotrope score (IS) was calculated using the following formula: dopamine dose (mcg/kg/min) + dobutamine dose (mcg/kg/min) + 100 × epinephrine dose (mcg/kg/min). VIS was calculated using the following formula: IS + 10 × milrinone dose (mcg/kg/min) + 10,000 × vasopressin dose (units/kg/min) + 100 × norepinephrine dose (mcg/kg/min) [14]. SNAP-II was calculated from six physiologic parameters, including lowest mean blood pressure, lowest body temperature, lowest PaO_2_/FiO_2_ ratio, lowest serum pH, presence of multiple seizures, and urine output. The sum of points assigned to each item was used to determine the final score [15]. Acute kidney injury (AKI) was defined using one of the following: an increase in serum creatinine by ≥0.3 mg/dL within 48 h; an increase in serum creatinine to ≥1.5 times the baseline, which is known or presumed to have occurred within the previous 7 days; or urine volume < 0.5 mL/kg/h for 6 h [16]. Small for gestational age (SGA) was defined as a birth weight of less than the 10th percentile for gestational age.

### 2.4. Management for PPHN

All patients were managed following NICU protocols for patients with PPHN. The NICU protocol included two options for ventilation management: conventional mechanical ventilation (CMV) and high-frequency oscillatory ventilation (HFOV). Mechanical ventilation was adjusted based on arterial blood gas results. Inotropic agents were adjusted to maintain systolic blood pressure or mean arterial pressure at the 50th percentile for the gestational age. iNO treatment was introduced to patients with OI > 20–25 with a starting dose of 20 parts per million. Pulmonary vasodilators were considered in patients who did not respond well to iNO therapy and those with moderate respiratory failure (OI > 15) who did not respond to supportive treatment. The type of pulmonary vasodilators depended on the attending physician’s decision [17]. The criteria for ECMO therapy were persistent severe hypoxia (OI > 40 or PaO_2_ < 40 mmHg) and persistent metabolic acidosis with refractory hypotension [18].

### 2.5. Statistical Analysis

Data were collected using EpiData and analyzed using R Program version 3.3.1 (R Foundation for Statistical Computing, Vienna, Austria). Continuous variables were presented as mean ± standard deviation (SD) and median (interquartile range [IQR]). Nominal variables were presented as numbers and percentages. To compare survivor and non-survivor groups, we used Fisher’s exact test and Wilcoxon rank-sum test for categorical variables, while Student’s *t*-test or Wilcoxon rank-sum test was used for continuous variables. To assess mortality-associated risk factors, the univariate and multivariate Cox’s proportional hazard models were used. We used a Kaplan–Meier survival curve with log-rank test to determine the survival rate at different time intervals. Statistical significance was set at *p* < 0.05.

## 3. Results

Between January 2010 and October 2023, 278 infants were diagnosed with PPHN, of whom 233 infants met the inclusion criteria. We excluded 52 infants because of chromosomal or multiple anomalies (*n* = 13), congenital heart disease (*n* = 19), and CDH (*n* = 13).

A total of 233 infants met our criteria, of which 198 (85%) and 35 (15%) were inborn and outborn, respectively. Table 1 summarizes the baseline characteristics of the neonates. There was no significant difference between survivors and non-survivors in terms of maternal factors, including pregnancy-induced hypertension, chorioamnionitis, and gestational diabetes.

PPHN was diagnosed based on clinical criteria or echocardiography. The median (IQR) age at diagnosis was 17 (8, 28) h. In our study, 211 (90.5%) patients underwent echocardiography. The overall median (IQR) right ventricular systolic pressure (RVSP) was 52 (41, 65) mmHg. Between survivors and non-survivors, echocardiography parameters, including RVSP, continuous right-to-left shunt to FO or DA, bidirectional shunt, and septal flattening were not significantly different. The overall median (IQR) baseline OI was 30.2 (17, 46.8). Of the 233 infants, 94 (40.3%) received iNO therapy with a median (IQR) duration of 60 (37, 93.5) h. All infants were administered inotropic agents and ventilated with HFOV. During the study, 47 (20.2%), 86 (36.9%), and 49 (21%) patients received sildenafil, bosentan, and milrinone, respectively.

The median (IQR) SNAP-II in all patients were 21 (10, 25), 16 (6, 23), and 16 (10, 28) at 0–12, 12–24, and 24–48 h of age, respectively. The median (IQR) maximum VIS at 24, 48, and 72 h of age were 75 (15, 230), 190 (60, 413) and 165 (30, 330), respectively.

**Figure 1:**
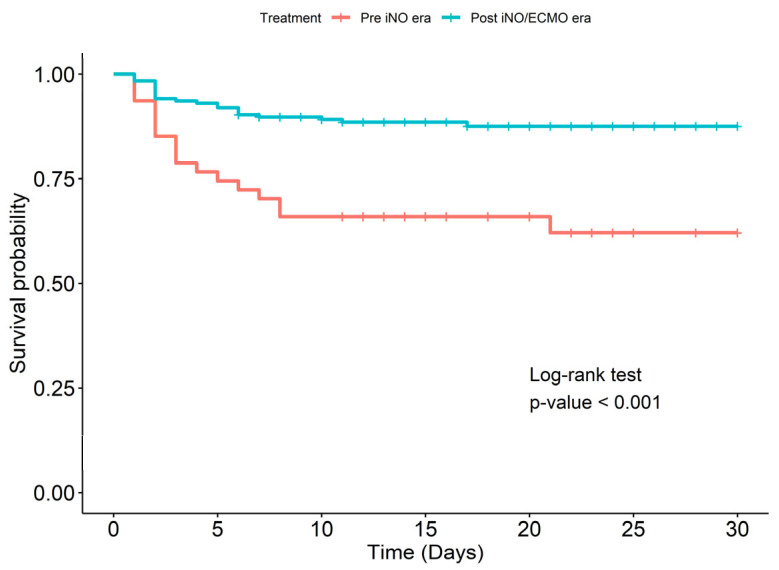
Kaplan–Meier survival curves comparing infants in the Pre-iNO era and the Post-iNO/ECMO era.

The overall in-hospital survival rate was 82.4% (192/233). The cumulative survival rates in all infants at the end of the 5th, 10th, and 30th days were 88.4% (95% CI: 84.4–92.6), 84.5% (95% CI: 79.9–89.3), and 82.2% (95% CI: 77.1–87.6), respectively. The survival rate during the period without iNO therapy (Pre-iNO era: 2010–2012) was 63.8% (30/47). It increased to 87.1% (162/186) during the period with iNO and ECMO therapy (Post-iNO/ECMO era: 2013–2023) (*p* < 0.001). The cumulative survival rates in infants with PPHN in the Pre-iNO era at the end of the 5th, 10th, and 30th days were 74.5% (95% CI: 63.0–88.0), 66% (95% CI: 53.7–81.0) and 62.1% (95% CI: 49.0–78.7), respectively. In the Post-iNO/ECMO era, the cumulative survival rates at the end of the 5th, 10th, and 30th days were 91.9% (95% CI: 88.1–95.9), 89.2% (95% CI: 84.8–93.8) and 87.5% (95% CI: 82.7–92.6), respectively (Figure 1).

**Figure 2:**
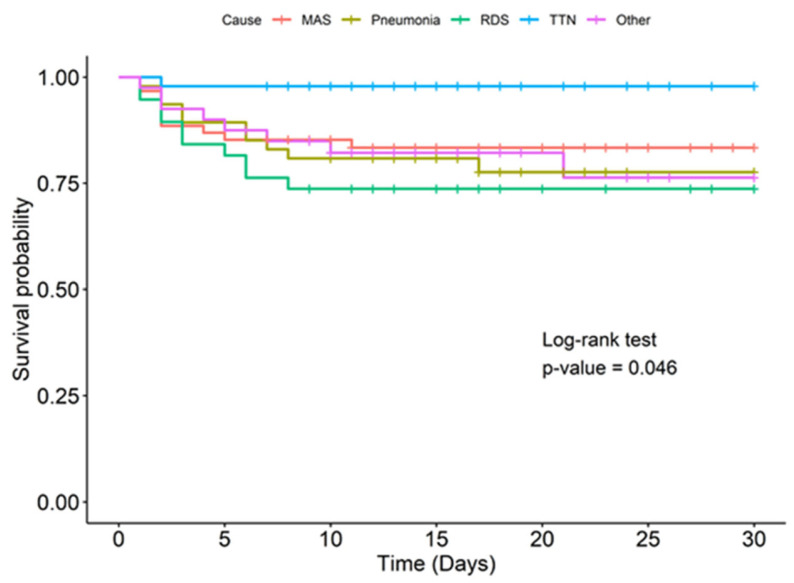
Kaplan–Meier survival curves in neonates with PPHN according to the etiology of PPHN.

At the end of the 30th day of life, the survival rates for infants with PPHN due to TTN, MAS, pneumonia, and respiratory distress syndrome (RDS) were 97.9% (95% CI: 93.8–100), 83.4% (95% CI: 74.5–93.4), 77.6% (95% CI: 66.1–91.1), and 73.7% (95% CI: 60.9–89.1), respectively (Figure 2).

Table 2 shows the survival rate and cumulative survival rates at the 5th, 10th, and 30th day of life in infants with and without iNO/ECMO therapy. We divided the patients into 4 groups according to OI level and whether they received iNO/ECMO treatment; (1) OI < 20 without iNO, (2) OI > 20 without iNO, (3) OI > 20 with iNO, and (4) OI > 40 with iNO/ECMO. The survival rate differed among the four groups (*p* = 0.004). The Kaplan–Meier survival analysis demonstrated a significant difference in the survival rate among the four groups (*p* = 0.046).

There were seven infants who did not respond to iNO and received ECMO therapy, and seventy-three infants who met the criteria for ECMO but for whom ECMO was not available. The survival rate was not significantly different between the two groups (85.7% vs. 67.1%, *p* = 0.42).

SGA was diagnosed in 19/233 (8.2%) infants. The SGA infants had a higher mortality rate than the non-SGA infants (82.9% vs. 17.1%, *p* < 0.001). Other outcomes including AKI, pneumothorax, duration of mechanical ventilation, and LOS were not significantly different between the groups.

AKI was diagnosed in 53 (22.7%) infants. The incidence of AKI in infants diagnosed with PPHN during the Pre-iNO era was higher than the Post-iNO/ECMO era (40.4% vs. 18.3%, *p* = 0.002). The risk factors for mortality were GA less than 28 weeks, SGA, AKI, pneumothorax, SNAP-II at 12–24 h ≥ 43 and VIS at age 24 h (Table 3).

The median (IQR) overall duration of mechanical ventilation and LOS were 7 (4, 15) days and 16 (10, 25) days, respectively. In the survivor group, the median (IQR) duration of mechanical ventilation, oxygen days, and LOS were 8 (5, 15.5), 11 (7, 19) and 18 (13, 28) days, respectively. Of the 192 survivors, 184 (95.8%) were screened for hearing impairment using otoacoustic emission or auditory brainstem response tests; 5 patients (2.7%) failed.

## 4. Discussion

Our study demonstrated that the risk of mortality in patients with PPHN increased with GA less than 28 weeks, SGA, AKI, pneumothorax, high VIS at 24 h of age and SNAP-II score at 12–24 h ≥ 43. The overall in-hospital survival rate in the 14-year study was 82.4%. The survival rate in the Post-iNO/ECMO era was higher than in the Pre-iNO era.

Our study is the first to identify an association between SGA and PPHN-related mortality. Previous research has demonstrated an association between SGA and the development of PPHN [6], as well as increased risk of post-discharge mortality or hospital readmission during the first year of life [19]. Growth restriction in utero can arise from various etiologies and may result in altered physiologic and metabolic functions in affected neonates, contributing to increased vulnerability to multiple morbidities [6,19]. The association between SGA and PPHN may be related to pulmonary artery endothelial cell dysfunction and impaired pulmonary alveolar and vascular growth [20], which may predispose to increased pulmonary vascular resistance and reduced adaptation to extrauterine life. These pathophysiologic alterations may explain the increased incidence of PPHN and the higher mortality risk observed in SGA infants with PPHN. Recognizing SGA as a high-risk feature can assist clinicians in anticipating complications, tailoring respiratory and hemodynamic management, and emphasizing the importance of close monitoring both during hospitalization and post-discharge.

In very preterm infants, impaired angiogenesis during lung development may disrupt alveolarization and contribute to both structural and functional abnormalities. Moreover, postnatal pulmonary vascular disease may lead to elevated pulmonary vascular resistance and early onset pulmonary hypertension [21]. Previous research reported early pulmonary hypertension in preterm infants associated with a higher risk of mortality before 36 weeks postmenstrual age or the development of BPD-associated pulmonary hypertension [22]. Our findings similarly indicate that infants born at less than 28 weeks’ gestation who developed PPHN had an increased risk of mortality.

AKI is common among NICU patients. AKI incidence varies with gestational age [23]. AKI is prevalent in 34%, 29.5%, and 28.4% of infants with PPHN in Egypt, Turkey, and Thailand, respectively [24,25,26], consistent with our findings.

Pneumothorax may occur as a complication of the resuscitation process, ventilatory strategies involving high positive pressure, or underlying conditions such as meconium aspiration syndrome (MAS). We found that pneumothorax was associated with an increased risk of mortality, consistent with previous studies, which reported that pneumothorax significantly increased the odds of mortality in PPHN (odds ratio = 5.27) [27].

Higher OI levels indicate greater PPHN severity [12]. iNO therapy is generally administered in patients with an OI > 20–25, whereas ECMO is indicated in neonates with an OI of >40 [28]. An OI > 40 is associated with mortality in infants with PPHN [10,16]. Our study showed that OI greater than 40 was associated with an increased risk of mortality in univariate analysis; however, this association was not statistically significant in the multivariate analysis.

SNAP-II helps predict neonatal mortality in the NICU [29]. An increased mortality risk is associated with a high SNAP-II [30]. A SNAP-II of 19 in the first 24 h of admission could predict death and/or the need for ECMO in infants with PPHN [31]. Infants with SNAP-II ≥ 43 have the highest mortality risk [32], which is similar to our findings.

VIS is useful for predicting treatment outcomes. The maximum VIS in non-survivors is significantly higher than that in survivors [33]. The maximum VIS during the 72 h after admission of 430 showed the good accuracy for predicting mortality in PPHN, with an AUC of 0.80 [18]. A VIS > 30 is associated with mortality in extremely low birth weight infants [34]. These findings, consistent with our results, demonstrate that high VIS can help predict neonatal mortality.

Other independent risk factors for mortality in infants with PPHN have been reported. Davis et al. [35] showed that the 5 min Apgar score, lowest pH, maximum ventilator rate, critical partial pressure of carbon dioxide, and highest inspiratory pressure are significantly different between survivors and non-survivors. Another study found that the risk factors for mortality are unresponsiveness to iNO therapy, a 1 min Apgar score ≤ 3, a low PaO_2_/FiO_2_ level, and a low pH level within 12 h after admission [36]. The risk factors associated with death in Asian countries are GA < 34 weeks, HFOV treatment with or without iNO, inotropic agents, and pneumothorax [11,37]. Referral patient, higher A-a gradient, and idiopathic PPHN were associated with death in another study [17]. In idiopathic PPHN, the presence of reversal of flow in the descending aorta and a 5 min Apgar score ≤ 5 are independent risk factors for mortality [38]. In our study, 5 min Apgar score < 5 was associated with increased risk of death; therefore, improvement of perinatal care and optimizing hemodynamics might help to improve outcomes and reduce mortality.

The mortality rate in infants with PPHN varies globally. There is a lower mortality rate (7–9%) in United States where iNO and ECMO are available [6,19]. Studies in Asian countries report mortality rates between 12% and 46.4% [11,39,40]. Our study reported a mortality rate of 36.2% from 2010 to 2012, which decreased to 12.9% after initiating iNO/ECMO therapy in our center (2013–2023).

We also compared the infants who required iNO in the Pre-iNO era to the infants who were treated with iNO in the Post-iNO/ECMO era. There were significant differences in survival rate and AKI rate between the groups; iNO therapy improved neonatal outcomes, including survival rate. ECMO has been available in our hospital since 2015. However, we only had seven infants who received ECMO therapy in our study, and we did not analyze the effect of ECMO on the survival rate of infants with PPHN. The other factors that improved the survival rate were the development of practice guidelines for PPHN management, ventilation optimization, and strategies for adjunctive therapy with pulmonary vasodilators.

We also observed that the survival rate was dependent on PPHN etiology. A higher survival rate was observed in PPHN infants with MAS, TTN, and pneumonia which corroborated previous studies [3,11]. Other neonatal outcomes observed in our study included duration of mechanical ventilation and hearing impairment. The median duration of mechanical ventilation in our study was 7 days, consistent with another study [11]. Hearing screening was performed before discharge from the hospital. Some studies have reported hearing impairment in 5.9–6.2% of survivors [41,42]; our study reported that 2.7% of survivors failed the hearing screening at discharge.

This study has limitations. First, this was a single-center retrospective study with a small sample size, and we included the period without iNO therapy in the analysis. Therefore, the overall results included survival rates, and factors associated with increased mortality risk should be carefully investigated in a center where iNO and ECMO are available. Second, we used the cut-off level of SNAP-II based on a previous study; future studies should investigate the best cut-off SNAP-II. Third, this study lacked long-term follow-up to evaluate morbidities after discharge.

A strength of this study is that 90% of total cases underwent echocardiography, the gold standard for diagnosing PPHN. Therefore, the diagnosis was accurate and reliable. Second, this is the first study to report an association between SGA and an increased risk of in-hospital mortality in infants with PPHN. Third, we analyzed the survival rates according to PPHN etiology and severity, which are useful details concerning infant outcomes. We showed the benefits of using SNAP-II, VIS and OI for assessing disease severity, which helps physicians in predicting the prognosis.

## 5. Conclusions

In this study, GA less than 28 weeks, SGA, AKI, pneumothorax, high VIS, and SNAP-II at age 12–24 h ≥ 43 were associated with an increased mortality risk. The improvement in survival rates was related to the provision of advanced care, including iNO and ECMO therapy in infants with PPHN.

## Figures and Tables

**Table 1:** Baseline characteristics of survivors and non-survivors with PPHN.

Baseline Characteristics	Non-Survivors (*n* = 41)	Survivors (*n* = 192)	*p*-Value
GA, weeks	37 (32, 38)	37 (35, 39)	0.047
GA < 28 weeks	10 (24.4)	5 (2.6)	<0.001
GA < 34 weeks	13 (31.7)	16 (8.3)	<0.001
Birth weight, g	2530 (1360, 3040)	2948 (2579, 3348)	0.002
SGA	7 (17.1)	12 (6.2)	0.052
1 min Apgar score	6 (3, 9)	8 (7, 9)	0.036
5 min Apgar score	8 (5, 9)	9 (8, 9)	<0.001
Age at PPHN diagnosis, h	14 (7, 24)	18 (9, 29.5)	0.243
RVSP, mmHg	55 (45, 65)	51.5 (40, 65)	0.511
OI at baseline	47.3 (33.1, 71.1)	26.9 (14.1, 42.2)	<0.001
MAP at baseline, cmH_2_O	15 (13.8, 17)	13 (11, 15)	<0.001
Cause of PPHN, *n* (%)			
MAS	10 (24.4)	51 (26.5)	0.927
Pneumonia/sepsis	12 (29.3)	38 (19.8)	0.346
TTN	1 (2.4)	46 (24)	0.004
RDS	11 (26.8)	27 (14.1)	0.076
Others	7 (17.1)	30 (15.6)	0.99
SNAP-II at age 12–24 h	31 (16, 37)	16 (5, 21)	<0.001
SNAP-II at age 24–48 h	31.5 (28, 41.8)	16 (5, 23)	<0.001
VIS at age 24 h	172 (22, 330)	65 (15, 219)	0.019
VIS at age 48 h	430 (230, 440)	149.5 (53.8, 315)	<0.001
Acute kidney injury, *n* (%)	19 (46.3)	34 (17.7)	<0.001
Pneumothorax, *n* (%)	15 (36.6)	37 (19.3)	0.027

Data are expressed as *n* (%), median (IQR) or mean ± standard deviation (SD). GA, gestational age; MAS, meconium aspiration syndrome; MAP, mean airway pressure; OI, oxygen index; PPHN, persistent pulmonary hypertension of the newborn; RDS, respiratory distress syndrome; RVSP, right ventricular systolic pressure; SGA, small for gestational age; SNAP, score for neonatal acute physiology; TTN, transient tachypnea of the newborn; VIS, vasoactive-inotropic score.

**Table 2:** The survival rate and cumulative survival rates at the 5th, 10th and 30th day of life in infants with and without inhaled nitric oxide and ECMO therapy.

	OI < 20 Without iNO (*n* = 56)	OI > 20 withoEut iNO (*n* = 83)	OI > 20 with iNO (*n* = 87)	OI > 40 with iNO and ECMO (*n* = 7)	*p*-Value
Survival rate, *n* (%)	54, (96.4)	60 (72.3)	72 (82.7)	6, (85.7)	0.004
Cumulative survival rates at the day of life, % (95% CI)					0.042
5th day	96.5 (91.3–100)	81.9 (74.0–90.5)	90.8 (84.1–96.9)	100 (100–100)	
10th day	95.7 (90.1–100)	77.8 (68.8–86.5)	83.9 (76.6–92.8)	100 (100–100)	
30th day	95.7 (90.1–100)	74.9 (65.8–85.3)	82.8 (74.8–91.7)	80 (51.6–100)	

ECMO, extracorporeal membrane oxygenation; iNO, inhaled nitric oxide; OI, oxygen index.

**Table 3:** Risk factors for predicting mortality in infants with PPHN.

Variable	Univariate		Multivariate	
	HR (95% CI)	*p*-value	Adjusted HR (95% CI)	*p*-Value
GA < 28 weeks	5.54 (2.62–11.7)	<0.001	5.46 (2.25–13.24)	<0.001
GA < 34 weeks	3.54 (1.79–6.99)	<0.001	–	–
BW < 2500 g	3.18 (1.69–5.96)	<0.001	–	–
SGA	2.72 (1.2–6.16)	0.032	2.93 (1.24–6.92)	0.026
5 min Apgar score < 5	4.21 (2–8.9)	0.001	–	–
OI ≥ 40	3.87 (2–7.49)	<0.001	–	–
Acute kidney injury	3.98 (2.08–7.60)	<0.001	2.48 (1.27–4.84)	0.01
Pneumothorax	2.13 (1.11–4.1)	0.03	3.03 (1.48–6.21)	0.003
SNAP-II at age 12–24 h ≥ 43	4.82 (1.98–11.73)	0.003	–	–
SNAP-II at age 24–48 h ≥ 43	12.76 (5.62–28.97)	<0.001	4.03 (1.66–9.77)	0.005
VIS at age 24 h	1.003 (1.001–1.005)	0.004	1.0026 (1.0004–1.005)	0.026
VIS at age 48 h	1.005 (1.003–1.008)	<0.001	–	–

BW, birth weight; CI, confidence interval; GA, gestational age; HR, hazard ratio; OI, oxygen index; SGA, small for gestational age; SNAP, score for neonatal acute physiology; VIS, vasoactive-inotropic score.

## Data Availability

The raw data supporting the conclusions of this study will be pro-vided available by the authors on request. The data are not publicly available due to privacy.
